# Basosquamous Cell Carcinoma: A Summary of the Definitions and Demographic, Clinical, Therapeutic, Histological, and Outcome Analysis of 20 Consecutive Basosquamous Cell Carcinomas in Comparison with 130 Basal Cell and 81 Squamous Cell Carcinomas in a Single Institution

**DOI:** 10.3390/jcm15062449

**Published:** 2026-03-23

**Authors:** En Hyung Kim, Chang Gok Woo, Eui-Tae Lee

**Affiliations:** 1 Department of Dermatology, College of Medicine, Chungbuk National University, Chungbuk National University Hospital, Cheongju 28644, Chungbuk, Republic of Korea; ehk@chungbuk.ac.kr; 2 Department of Pathology, College of Medicine, Chungbuk National University, Chungbuk National University Hospital, Cheongju 28644, Chungbuk, Republic of Korea; thewallflower@chungbuk.ac.kr; 3 Department of Plastic and Reconstructive Surgery, College of Medicine, Chungbuk National University, Chungbuk National University Hospital, Cheongju 28644, Chungbuk, Republic of Korea

**Keywords:** basosquamous cell carcinoma (BSC), definitions, basal cell carcinoma (BCC), squamous cell carcinoma (SCC), wide local excision (WLE), local flaps, Kaplan–Meier survival analysis, log-rank test, Cox proportional hazards regression analysis

## Abstract

**Objectives**: To clarify the characteristics of Basosquamous cell carcinoma (BSC), this study compares demographic, clinical, therapeutic, histological, and outcome findings of BSCs with those of basal cell carcinoma (BCC) and squamous cell carcinoma (SCC). **Methods**: The authors classified various definitions of BSC into three groups: the broadest, modest, and narrowest definitions. This study adopted the narrowest definition (both BCC and SCC features with transition zones in between) due to its wide use, its adoption by the World Health Organization, and the least heterogeneous definition. From 2009 to 2018, 20 consecutive cases of BSC presented in a single institution, along with 130 cases of BCC and 81 cases of SCC. **Results**: The statistically different parameters of BSC compared to BCC or SCC were age (SCC > BSC, BCC), duration (BSC, BCC > SCC), unclear border (BSC > BCC, SCC), higher NCCN classification (BSC, SCC > BCC), safety margin (SCC > BSC > BCC), operation time (BSC, SCC > BCC), reconstruction (less primary closure in BSC than BCC), microscopic size (BSC, SCC > BCC), perineural invasion (BSC > BCC), free lateral margin (BSC, SCC > BCC), and follow-up period (BSC > BCC, SCC). Regarding outcome, one distant metastasis (6.3%) in BSCs, no aggressive consequences in BCCs, and four local recurrences (11.1%), two lymph node metastases (5.6%), and one distant metastasis (2.7%) in SCCs were observed. **Conclusions**: In this Asian cohort, BSC has a trend toward higher rates of overall adverse outcomes compared to BCC, although this difference did not reach definitive statistical significance, unlike the findings reported in Caucasian populations. Early detection and appropriate treatment at the individual patient level are warranted to minimize rare but clinically relevant adverse events and reproduce favorable outcomes at the population level. Wide local excision followed by local flaps could be a successful surgical option with an adequate safety margin and double histopathologic intraoperative and postoperative check-up.

## 1. Introduction

Basosquamous cell carcinoma (BSC) is a rare, potentially aggressive cutaneous tumor consisting of both basal cell carcinoma (BCC) and squamous cell carcinoma (SCC) components with an intervening transition zone of intermediate (or metatypical) cells. Due to its rarity and heterogeneous composition, there have been many controversies regarding its existence, pathogenesis, prognosis and treatment [1,2,3,4,5]. In particular, as an intermediate form, BSC has been associated with various definitions and potential misdiagnoses. The authors categorized these definitions into three groups and adopted the narrowest definition for this review and original study.

The authors implemented risk factors of the American Joint Committee on Cancer staging eighth edition (AJCC-8) [6,7], National Comprehensive Cancer Network staging (NCCN) [8,9] and Brigham and Women’s Hospital (BWH) staging system [10] in a retrospective analysis of 20 consecutive BSC cases between 2009 and 2018. Detailed demographic, clinical, therapeutic, histological and outcome parameters were employed.

This case–control study included 130 BCC and 81 SCC cases during the same time period as control groups to compare the biological characteristics and relative aggressiveness of the three cancers.

In non-Caucasian populations, only a few cases—mainly case reports—have been published in English or Korean [11,12,13,14,15,16,17]. This study represents the first case series with long-term follow-up in Asian patients, enabling an indirect comparison with previous BSC reports in Caucasian populations (Supplementary Table S1).

It is also the first case–control study for BSC in which Kaplan–Meier survival analysis was applied with the log-rank test and Cox regression analysis.

## 2. Materials and Methods

### 2.1. Categorization of Three Definitions (Table 1)

Many debates about BSC stem from discordances in definition. Therefore, to begin this study, the authors decided to review and categorize the territory of BSC and distinguish it from other related confusing terms and tumors [1,3,18]. There have been at least three definitions of BSC in the literature.

The broadest definition of BSC is provided by the WHO in 2005 as follows: “tumor with infiltrative growth, with areas of keratinization and/or intercellular bridge formation, in the setting of a prototypic proliferative stromal reaction.” [4,19,20,21]. Its modest definition coincides with metatypical basal cell carcinoma (MBCC) as a tumor composed of metatypical (intermediate) cells with or without a BCC component but without a typical SCC component [18,22,23].

The narrowest definition of BSC is as follows: carcinomas with areas of both basaloid and squamoid cells linked by a transition zone with intermediate (metatypical) cells of mixed immunohistochemical pattern with Ber-EP4 (or Moc 31) and epithelial membrane antigen (EMA) [1,2,24,25,26,27,28,29,30,31,32,33,34].

In some older studies, the definition takes both basaloid and squamoid cells but fails to mention the transition zone, presence of intermediate cells, and immunohistochemical findings [35,36,37,38,39]. They are recognized as the narrowest definition variant. The definitions of BSC are summarized in Table 1.

**Table 1:** Categorization of BSC definitions, their applications and outcomes in the literature ＊.

Definitions †	Reference/Authors, Year ‡	Type of Study #	Patients $	Control No. ∫	Tx Methods ∬	Local	LNM	DM	F/U, Months
			No.(F/U)	& type		recurrence			mean (range)
Narrowest	WHO, 2022 [25]								
	Lee ET et al., 2026 ^∆^	Retrospective CCS	20	130 BCCs, 81 SCCs	WLE	0%	0%	6.3%	96.0 (1–182)
	Gualdi G et al., 2021 [1]	Prospective CCS	288	5754 KTs	SMART approach				0
	Kakagia DD et al., 2018 [26]	Prospective CCS	142	SLNB (+) vs. (−)	WLE + SLNB ± PD		1.4%	0%	52 (18–84)
	Akay BN et al., 2017 [24]	Retrospective CS	36			0%	0%		
	Bucci T et al., 2016 [27]	Retrospective CR	2		MMS + LND ± PD, ART	2/2	2/2	0/2	12, 36
	Wermker K et al., 2015 [28]	Retrospective CS	89		MMS	4.5%	5.6%	2.2%	48 (12–112)
	Kececi Y et al., 2015 [29]	Retrospective CCS	35	1647 NMSCs		4%	0%	0%	87 (12–168)
	Yoshida Y et al., 2013 [11]	Retrospective CR	1		WLE + ART + CTx	1/1	1/1		20
	Lima NL et al., 2012 [30]	Retrospective CR	1		WLE	0/1	0/1	0/1	6
	Mougel F et al., 2012 [31]	Retrospective CCS	12 OTR	30 (non-OTR)	surgical excision	0%	0%	0%	34
	Volkenstein S et al., 2010 [32]	Retrospective CR	1		WLE	0/1	0/1	0/1	12
	Leibovitch I et al., 2005 [33]	Prospective CS	178		MMS	4.1%	2%	0%	>60
Narrowest variant	Venturi F et al., 2024 [40]	Retrospective CS	192						
	Bowman PH et al., 2003 [35]	Retrospective CCS	27	973 NMSCs	MMS			7.4%	cross-sectional
	Martin RC et al., 2000 [36]	Retrospective CS	28			26%	18%	3.6%	60 (12–312)
	Johnson BF et al., 1989 [37]	Retrospective CR	3		RT 2, no Tx 1		2/3		54–132
	Schuller DE et al., 1979 [38]	Retrospective CCS	33	2532 NMSCs	WLE	12%	6.1%		
	Borel DM et al., 1973 [39]	Retrospective CCS	32	1671 NMSCs	WLE ± ART	46%	9.3%	0%	82
Modest	Allen KJ et al., 2014 [18]	Retrospective CCS	160	131 BCCs	MMS	3.8%	0%	0%	58
	Tarallo M et al., 2008 [22]	Retrospective CS	240		WLE + ART + CTx	10%	1.6%	0%	60
	Silistreli OK et al., 2006 [23]	Retrospective CS	35		WLE ± ART, CTx	31%	14%	3%	18 (6–36)
Broadest	WHO, 2005 [20]								
	Betti R et al., 2013 [19]	Retrospective CCS	76 (35)	3948 NMSCs	WLE	5.7%	0%	2.9%	60
	Skaria AM et al., 2010 [21]	Retrospective CCS	56	944 NMSCs	MMS	8.9%	0%	0%	60 (24–129)
	Garcia C et al., 2009 [4]	Narrative review				4–51%	5%		0–360
Not specified	Zhu GA et al., 2014 [41]	Retrospective CS	19		WLE &/or ART, CTx		37%	53%	35 (16–50)

＊ LNM, lymph node metastasis; DM, distant metastasis; F/U, follow-up. † The narrowest definition means carcinomas with areas of both basaloid and squamoid cells, linked by a transition zone with intermediate (metatypical) cells of a mixed immunohistochemical pattern with Ber-EP4 (or Moc 31) and epithelial membrane antigen (EMA) as defined by WHO in 2022. The narrowest variant definition does not refer to a transition zone and/or metatypical (intermediate) cells. The modest definition coincides with metatypical basal cell carcinoma (MBCC) as a tumor composed of metatypical (intermediate) cells with or without a BCC component but without a typical SCC component. The broadest definition means a tumor with infiltrative growth, with areas of keratinization and/or intercellular bridge formation, in the setting of a prototypic proliferative stromal reaction as defined by WHO in 2005. ‡ Zhu GA et al., 2014 [41], provided an analysis of stage 4 disease (at least T4, N2, or M1) according to AJCC-7 guidelines. # CCS, case–control series; CS, case series; CR, case report. $, total numbers of patients (numbers in parenthesis represent numbers of patients followed up for a given time); OTR, organ transplant recipient. ∫ KT, keratinizing tumors; SLNB, sentinel lymph node biopsy; NMSC, non-melanoma skin cancer. ∬ Tx, treatment; SMART, superficial margin assessment by reflectance confocal technique; WLE, wide local excision; PD, parotidectomy; MMS, Mohs micrographic surgery; LND, lymph node dissection; ART, adjuvant radiation therapy; CTx, chemotherapy; RT, radiation therapy. ^∆^ This study.

**Figure 1:**
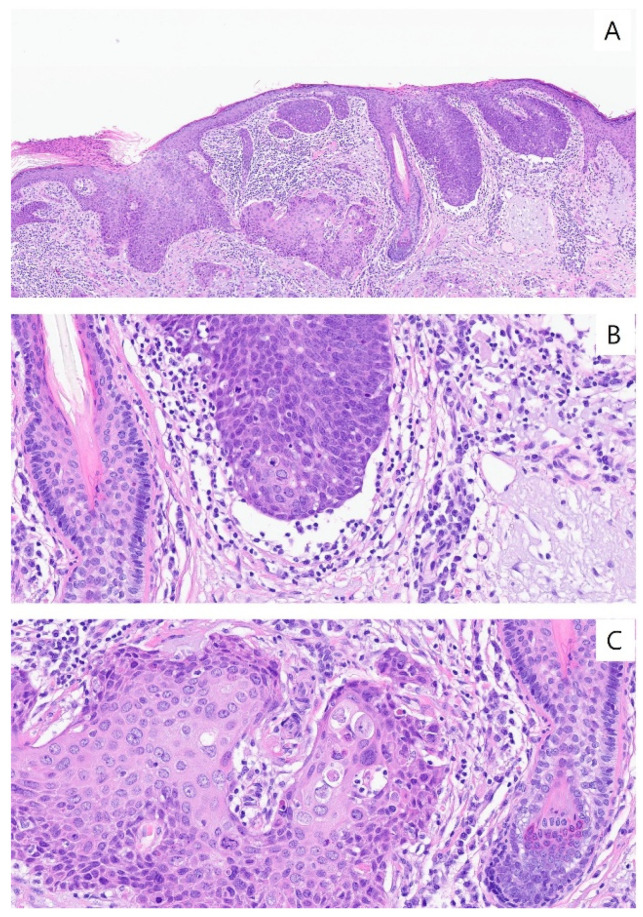
The narrowest definition of basosquamous cell carcinoma. (**A**) The tumor shows two carcinoma components with both basaloid (**right upper**) and squamous differentiation (**left lower**). Two carcinomas are linked by a transition zone with intermediate (metatypical) cells. Epidermal attachment is variably present (HE × 100). (**B**) Large basaloid lobules with peripheral nuclear palisade and cleft formation between tumor lobules and stroma are observed in basal cell carcinoma (HE × 400). (**C**) Pleomorphism is generally mild and mitotic activity is variable. In contrast, squamous cell carcinoma does not have any peripheral palisading or clefting. Along with nuclear pleomorphism, typical squamous differentiation, such as keratinization and intercellular bridges, is easily recognizable (HE × 400).

In this study, the narrowest definition of BSC was adopted because it is the most commonly used form in the literature, compatible with the most recent WHO criteria in 2022, and with the intention of using strict inclusion criteria to collect the least heterogeneous group of tumors possible for a thorough comparative study (Figure 1).

### 2.2. Differential Diagnoses

Differential diagnosis lists include collision tumor, keratinizing BCC, BCC with squamous differentiation, and metatypical basal cell carcinoma (MBCC). A collision tumor has separate areas of BCC and SCC, but no transition zone linking them, and each BCC and SCC component has a distinct and independent origin in the epidermis. Keratinizing BCC contains abrupt keratinization usually at the center of the BCC tumor nodule, but lacks intervening areas of squamous cells or metatypical cells and the densely collagenized, fibroblast-rich stroma of an aggressively growing BCC [3,4]. BCC with squamous differentiation, which usually has a small squamoid portion at the center of the tumor nodule, is relatively common. However, similar to keratinizing BCC, it lacks metatypical cells and invasive SCC [14,18]. In accordance with the narrowest definition, the authors excluded collision tumors, keratinizing BCC, BCC with squamous differentiation and MBCC from this study.

### 2.3. Patients

From June 2009 to January 2018, 20 consecutive cases of BSC were identified at a tertiary hospital (Chungbuk National University Hospital). The medical records during the same eight and half-year time span presented 20 BSCs in 20 patients, 130 BCCs in 120 patients and 81 SCCs in 68 patients. One BSC patient also had a concomitant SCC, and BCC and SCC were presented together in three patients. According to the 2022 WHO criteria, only lesions with both BCC and SCC features, as well as transition zones between the two, were selected as BSC (Figure 1) [25]. Two or more non-melanoma skin carcinoma (NMSC) tumors in one patient were counted as many-tumor cases except for seven demographic (age, gender) and outcome parameters (follow-up more than 60 months, local relapse, lymph node metastasis, distant metastasis and total number of adverse outcomes). As exclusion criteria, other skin cancers, such as melanoma, melanoma in situ, lymphoma, adnexal cancers, dermal sarcomas, and metastatic carcinomas were excluded from the study.

For each patient with NMSC, demographic, clinical, histological, therapeutic and outcome parameters were collected in detail as follows: 1. Demographic parameters: age, gender. 2. Clinical parameters: location, duration, previous treatment (primary vs. recurrent cases), size (clinical), gross morphology (border demarcation, pearly appearance, pigmentation, erythema, ulcer), clinical T staging on TNM classification according to the American Joint Committee on Cancer (AJCC-8) [6,7], low-risk versus high-risk versus advanced or very-high-risk lesions according to the National Comprehensive Cancer Network (NCCN) classification [8,9], Brigham and Women’s Hospital (BWH) T staging [10], immunosuppression. 3. Therapeutic parameters: safety margin, number of resection for negative frozen results at the first operation, total number of operations needed for tumor eradication, operation time in minutes, method of anesthesia, reconstruction method, adjuvant therapies. 4. Histological parameters: initial diagnosis on preoperative punch biopsy, size (pathological), tumor thickness in mm, invasion depth in relation to the skin (maximal vertical infiltration = MVI; S = superficial: dermis; M = medium: subcutaneous fat; D = deep: fascia, muscle, cartilage, or bone), Clark level, perineural and lymphovascular invasion, R classification (resection status: R0 = negative margins; R1 = microscopically positive margins; R2 = macroscopically positive margins), free lateral and deep margin in mm, pathological T staging on TNM classification according to the AJCC-8 [6,7]. 5. Outcome parameters: follow-up period, the number of patients followed by for more than 60 months, local relapse, lymph node metastasis, distant metastasis and total number of adverse events. Data were compared between the BSC, BCC, and SCC groups to provide a statistical analysis of relative biological behaviors (Table 2, Table 3, Table 4, Table 5 and Table 6).

### 2.4. Surgical Technique

As surgical techniques, all epithelial cancers were treated with wide local excision (WLE), intraoperative frozen section control (IOFS) and postoperative permanent section control (POPS). The safety lateral margin was preoperatively determined from the initial histopathologic diagnosis of a single-sample punch biopsy performed either at the authors’ office or referral clinics, as 3–4 mm for low-risk BCC, 6 mm for high-risk BCC and low-risk SCC, and 10–20 mm for high-risk SCC [7,8]. During the treatment period of this study, between June 2009 and January 2018, the NCCN guidelines for NMSC did not incorporate the classification of advanced or very-high-risk lesions.

### 2.5. Statistical Analysis

This study was approved by the internal review board of Chungbuk National University Hospital (approval no. 2019-03-019). All statistical analyses were conducted using SPSS version 31 (SPSS Inc., Chicago, IL, USA). Differences in the continuous and ordinal variables among the three groups were assessed using the Kruskal–Wallis test. Nominal variables were compared among three groups using Fisher’s exact test. When an overall significant difference was detected (*p* < 0.05), pairwise comparisons were conducted using the Mann–Whitney U test for continuous and ordinal variables and pairwise Fisher’s exact test for nominal variables as post hoc analyses. To account for multiple comparisons, the Bonferroni correction was applied, and statistical significance for pairwise tests was set at *p* < 0.017 (0.05/3).

Kaplan–Meier analysis with the log-rank test was used to estimate disease-free survival outcomes among the three groups. When an overall significant difference was detected (*p* < 0.05), pairwise comparisons between two groups were performed using Cox proportional hazards regression, and statistical significance for these pairwise tests was set at *p* < 0.017 (0.05/3). Outcomes such as local recurrence, lymph node metastasis, and distant metastasis were considered as adverse events.

## 3. Results

### 3.1. Demographic Parameters

Twenty patients presented with 20 BSCs at a median age of 72.5 years (ranging from 50 to 90) and a male-to-female ratio of 1:1.5 (8 males and 12 females). The relative incidences of BSC, BCC and SCC among NMSCs were 8.7% (20/231), 56.3% (130/231) and 35.1% (81/231), respectively. The age of 68 SCC patients with 81 SCCs was significantly older than that of 20 BSC patients and 120 patients with 130 BCCs. The gender ratio of all the groups showed female dominance in common and was not significantly different (Table 2).

### 3.2. Clinical Parameters

The location of BSC was heavily focused (90.0%) on the head and neck, similar to BCC (91.5%) and SCC (85.2%). Durations of BSC (4.7 ± 3.5 years) and BCC (3.3 ± 4.9 years) were significantly longer than that of SCC (2.0 ± 2.2 years). The median clinical dimension of BSC was 12.5 mm with a large distribution range (6–110 mm), yet did not differ significantly from BCC (median 10.0 mm, range: 3–35 mm) and SCC (median 18.0 mm, range: 7–135 mm).

Regarding gross morphology, the ratio of ill-defined border (75.0%, 14.6%, 16.0%), pearly appearance (5.0%, 9.2%, 0%), pigmentation (70.0%, 69.2%, 38.3%), erythema (15.0%, 12.3%, 27.2%), and ulcer (25.0%, 10.8%, 22.2%) are given in parentheses in the order of BSC, BCC, and SCC. An ill-defined border favored BSC, while a pearly appearance was more indicative of BCC than SCC, and pigmentation was more common in BCC than SCC. Erythema favored SCC over BCC. These gross findings were statistically significant. However, in practice, a diagnosis of BSC had never been clinically suggested before biopsy.

Regarding clinical staging according to the AJCC-8th edition, cT1, cT2 and cT3 accounted for 83.3%, 11.1% and 5.6% of BSC, respectively; however, this was not statistically significant compared with BCC and SCC. According to the NCCN classification, most BSCs (15 patients, 75.0%) were classified as in the high-risk group, and five patients (25.0%) were classified as very-high-risk. On BWH T staging, fourteen, three, and three patients (70.0%, 15.0%, and 15.0%) were in the T1, T2a, and T2b stages, respectively (Table 3). BSCs presented with higher NCCN risk categories than BCCs. SCCs showed higher cT stages and NCCN classification than BCCs.

### 3.3. Therapeutic Parameters

The safety margin for BSC was 6.6 ± 3.9 mm (range: 3.0–20.0, median 5.0). This was wider than BCC (4.4 ± 1.3 mm) but narrower than SCC (8.5 ± 6.1 mm). The mean operation time for BSC (77.5 ± 34.9 min) and SCC (96.8 ± 65.6 min) was significantly longer than BCC (68.5 ± 34.3 min).

Most operations (18/20, 90.0%) were performed under local anesthesia with or without intravenous anesthesia, except for the two largest cases sized 110 and 75 mm (85 and 46 mm pathologically), under general anesthesia.

As reconstruction methods, less primary closure was done in BSC than in BCC (Table 4).

### 3.4. Histological Parameters

Preoperative one-sample punch biopsy at the office revealed twelve BCCs, one actinic keratosis, two SCCs, and five BSCs as the initial diagnoses. The initial misdiagnosis rate (false negative rate) was 75.0% (15/20) prior to permanent biopsy.

The maximal histological dimensions of both BSC (median 10.0 mm, range: 2–85 mm) and SCC (median 10.8 mm, range: 1–130 mm) were significantly larger than BCC (median 6.0 mm, range: 0.3–32 mm).

Perineural, lymphovascular and muscle invasion was detected in two, zero, and one patient in each category (10.0%, 0%, 5.0%), respectively, but without local relapse, lymph node metastasis and/or distant metastasis in these patients.

Free lateral and deep margin were 4.3 ± 2.9 (range: 1–14, median 4.0) and 2.3 ± 1.5 (range: 0.4–7, median 2.0) mm. Regarding pathological staging according to the AJCC-8, pT1, pT2 and pT3 accounted for 83.3%, 0% and 16.7% of BSC, respectively (Table 5).

### 3.5. Outcome Parameters

Median follow-up periods were 96.0 (range: 1–182), 54.0 (range: 1–190), and 40.0 months (range: 1–175) for BSC, BCC and SCC, respectively.

Fifteen of the 20 BSC patients (75.0%), sixty-one of the 120 BCC patients (50.8%), and thirty-three of the 68 SCC patients (48.5%) were followed up for more than a 60-month period. The reasons for the shorter follow-up of less than 60 months in patients with BSC were three follow-up losses and two deaths unrelated to BSC.

No local relapse or lymph node metastasis was observed in BSC patients. Only one BSC patient (1/16 = 6.3%) with the largest tumor of 85 mm size and 7 mm thickness pathologically (110 mm size clinically) on the back developed distant metastasis to the lung. His stage was pT3 on T staging of AJCC-8, very-high-risk group on NCCN classification, and T2b on BWH T staging. The patient was lost to follow-up at 22 months after surgery.

In BCC, no adverse outcome was observed. In four SCC patients, four local recurrences (4/36 = 11.1%), two lymph node metastases (2/36 = 5.6%), and one distant metastasis (1/36 = 2.7%) were identified at 1, 4, 6, and 30 months postoperatively. Among these four adverse outcome SCC cases, one patient with local relapse only without lymph node or distant metastasis was successfully treated with a second wide local excision and remained disease-free for 102 months. The other three patients with local recurrence, lymph node or distant metastases were lost to follow-up (Table 6).

**Figure 2:**
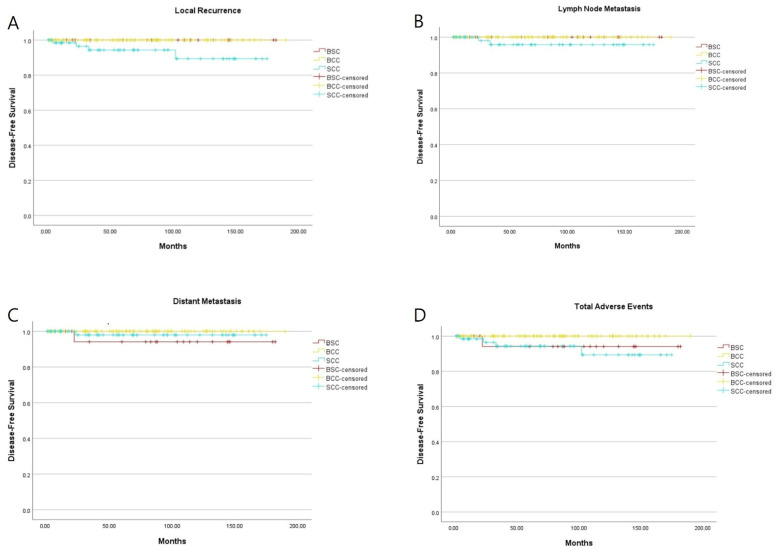
Kaplan–Meier analysis of disease-free survival. (**A**) Local recurrence (*p* = 0.019, log-rank test). (**B**) Lymph node metastasis (*p* = 0.131, log-rank test). (**C**) Distant metastasis (*p* = 0.134, log-rank test). (**D**) Total adverse events (*p* = 0.046, log-rank test). Tick marks indicate censored data. Statistical significances from the log-rank test for local recurrence and total adverse events were lost after multivariate adjustment on Cox regression analysis (BSC vs. BCC: *p* = 1.000 and *p* = 0.953 for local recurrence and total adverse events, respectively; BSC vs. SCC: *p* = 0.629 and *p* = 0.737, respectively).

On Kaplan–Meier analysis with the log-rank test for disease-free survival outcomes, comparisons among BSC, BCC and SCC yielded *p* values of 0.019, 0.131, 0.134, and 0.046 for local recurrence, lymph node metastasis, distant metastasis, and total adverse events, respectively (Figure 2). Consequently, significant differences were observed among the three groups for local recurrence and total adverse events. However, multivariable Cox proportional hazards regression revealed no significant differences for BCC or SCC versus BSC in these endpoints (BSC vs. BCC: local recurrence, *p* = 1.000, HR 1.003, 95% CI 0.000–9.87 × 10^9^; total adverse events, *p* = 0.953, HR 0.000, 95% CI 0.000–4.80 × 10^170^; BSC vs. SCC: local recurrence, *p* = 0.629, HR 165.595, 95% CI 0.000–1.68 × 10^11^; total adverse events, *p* = 0.737, 95% CI 0.162–13.155).

Statistical significance from the log-rank test was lost after multivariate adjustment via Cox regression analysis.

The number of censored cases for BSC, BCC, and SCC in the order of unrelated deaths, follow-up losses, and total censored cases were 3, 3, and 6 (15.0, 15.0, 30.0%) for BSC, 12, 46, and 58 (9.2, 35.4, 44.6%) for BCC, and 11, 27, and 38 (13.6, 33.3, 46.9%) for SCC, respectively.

Among the outcomes, a statistically significant parameter was the longer follow-up period for BSC over BCC and SCC. A trend toward more frequent total adverse events for BSC compared to BCC and a trend toward more frequent local recurrence and total adverse events for SCC compared to BCC were also found.

### 3.6. Summary of Results

In summary, one demographic (age), three clinical (duration, border, NCCN classification), three therapeutic (safety margin, operation time, reconstruction method), three histologic (microscopic size, perineural invasion, free lateral margin), and one outcome parameter (follow-up period) were significantly different in BSC compared with the other two types of NMSC. Among the outcome parameters, a trend toward more frequent total adverse events in BSC compared with BCC was also noted.

## 4. Discussion

It is clinically important to consider BSC in the differential diagnosis of NMSC. That is because misdiagnosis is common even after preoperative shave or punch biopsies, and potentially aggressive BSC could be mistaken for nonaggressive BCC [3,5]. In this study, the initial preoperative diagnoses included 12 BCCs, one actinic keratosis, two SCCs, and five BSCs on one-sample punch biopsy at the office. The sensitivity was only 25.0%, which is comparable with the 13.7% reported by Leibovitch (2005) and 52.8% reported by Allen (2014) [18,33]. Thus, nonoperative treatment modalities, such as electrodessication and curettage, topical agents, or cryotherapy in which permanent specimens could not be taken, should be reserved only for small superficial low-risk tumors in patients with serious comorbidities. Nonetheless, for BCC, electrochemotherapy (ECT), which combines electroporation with cystostatic agents, such as bleomycin, has emerged as a viable alternative for complex cases [42]. ECT is particularly beneficial for patients presenting with multiple lesions, tumors in surgically difficult locations, or comorbidities that contraindicate traditional surgery. This treatment mode could be considered for an elderly population with BSC.

The definition of BSC has evolved from the broadest categorization to the narrowest one. A few of the previous studies on BSC with the largest sample sizes comprised MBCC cases and, therefore, could not be directly compared or referenced with recent BSC studies including this study [18,22,23]. Various hybrid tumors between pure BCC and pure SCC, such as collision tumor, keratinizing BCC, BCC with squamous differentiation, and MBCC, should be carefully identified, differentiated from a true BSC and treated accordingly. A collision tumor should be treated as two separate cases of BCC and SCC. A keratinizing BCC and a BCC with squamous differentiation can be regarded as subtypes of BCC and unduly aggressive treatment should be avoided. MBCC may be regarded as a separate entity, and treatment should be carefully extrapolated from the treatment of SCC, similar to BSC [9,18,43]. Further investigation of the nature, clinical behavior and treatment of MBCC is warranted.

BSC has been reported to have various incidence rates ranging from 0.18% (1/560) of BCCs [44] to 14.3% (17/119) of all skin cancers (Montgomery, 1928) [5] in Caucasian populations. However, both Montgomery’s and Burston and Clay’s reports were published before the establishment of proper definitions of BSC. According to the narrowest definition adopted by this study, five reports presented 2.1% (35/1682) of NMSC [29], 2.1% of NMSC [45], 2.7% (27/1000) of Mohs micrographic surgery (MMS) patients [35], 4.7% of NMSC [40] and 4.8% (288/6042) of keratinizing tumors [1]. No incidence rate was reported according to the modest definition and 1.9% (76/4024) [19] and 5.6% (56/1000) [21] among NMSC cases were reported according to the broadest definition.

All reports found a predisposition to old Caucasian males [1,3,5,40]. However, ethnicity and community might play important roles in the results. As BCC and SCC are much less common in Asian populations than in Caucasian populations, BSC has also been reported much less frequently [46,47]. Until now, only 14 cases in 15 reports have been published, mainly in the form of nine case reports, in English or Korean languages, for non-Caucasian populations [11,12,13,14,15,16,17]. The cumulative incidence rates of BSC in those six original articles were 0.8% (3/362) of BCCs to 2.2% (2/92) of facial skin cancers (Supplementary Table S1) [12,13,15,16,17]. The incidence rates of MBCC in the same articles were 4.2% (9/213) [17] to 13.8% (11/87) of BCCs [15]. The incidence rates of BCC and SCC in the same articles range from 56.2% (41/73) to 57.9% (11/19) and from 23.3% (17/73) to 26.3% (5/19), respectively [12,13]. BSC is less common than MBCC in Korea, as inferred from its definition [18]. The relative incidences of BSC compared to NMSC in previous non-Caucasian studies were not apparently different from those in the Caucasian population [29,35]. However, in this study, the relative incidence of BSC compared to NMSC (8.7%, 20/231) was much higher than previous studies in Korea and in the Caucasian population. This is probably due to previous lack of interest, misdiagnosis and underreporting in previous studies, concentration of patients, sampling bias in the authors’ tertiary hospital, and the population characteristics of the authors’ community, which is heavily dominated by older people in rural areas.

In this study, the male-to-female ratio was 1:1.5 (8 to 12), 1:1.55 (47 to 73), and 1:1.96 (23 to 45) for BSC, BCC and SCC, respectively. Contrary to the male predisposition in the Caucasian population, this female predominance was also noted in other recent Korean studies on NMSC [46,47]. Most authors attributed this trait to female predominance in the older population because of their longer life expectancy.

Upon physical examination, BSC exhibited irregular borders more frequently. BSC (70.0%) and BCC (69.2%) patients shared high pigmentation rates, but only BCC had a significantly higher pigmentation rate (BSC vs. SCC: *p* = 0.044; BCC vs. SCC: *p* < 0.001) compared with SCC (38.3%) on pairwise Fisher’s exact test after Bonferroni correction at *p* < 0.017 (0.05/3). The high pigmentation rate of BSC in this study contrasts with around 5–15% in the Caucasian population [5]. A high incidence of pigmentation is also a known characteristic of the Korean BCC population [16].

Regarding clinical and pathological T staging, BSC were classified into more advanced stages than BCC only on NCCN classification. The AJCC-8 clinical T staging, BWT T staging, and AJCC-8 pathological T staging did not differ significantly between BSC and the other two tumors (Table 3 and Table 5).

However, along with a low incidence rate, there is practically no specific gross clinical finding that can differentiate BCC, SCC, and BSC among NMSCs, as indicated in this article and many other studies [5,19,29,38]. Thus, a final histologic examination of the surgical en bloc excision specimen is necessary to establish the correct diagnosis [5]. In particular, if a BCC has aggressive clinical features such as irregular borders, or necessitates multiple attempts to acquire clear frozen margins, attention should be paid to the possibility of hidden BSC components in the specimen. Recently, some trials have been conducted to distinguish between these tumors using dermoscopy and confocal microscopy by clinico-dermoscopico-confoco-pathological correlation [5,24,40].

Among therapeutic parameters, except for the intended wider safety margin according to the initial diagnosis, there was no significant difference in the initial resection attempts for free frozen margin and total number of operations for tumor eradication, which would reflect the potential aggressiveness of a tumor. These results matched with those of Allen’s report [18], with the same one Mohs’ layer requirement for complete removal in BCC, BSC and MBCC. However, this did not agree with Kececi’s result [29] of 31.4% of initial margin positivity in BSC. But, the latter study attributed this to misdiagnosis and the resultant narrow safety margin and obtained a local recurrence rate of 4% on re-excision after the first operation. Both studies did not report any regional or distant metastasis.

Significantly longer operation time and more general anesthesia were required for the SCC group than for the BCC group. However, except for longer operation time and less primary closure for BSC than for BCC, there was no statistical difference in therapeutic parameters between the BSC group and the other two groups. These results could indirectly reflect the intermediate biological behavior of BSC; however, small sample size and retrospective study design precluded confirmatory conclusions.

Contrary to recent NCCN guidelines, which recommend second intention healing, linear repair, or skin graft for reconstruction of both BCC and SCC [8,9], the authors primarily applied local flaps to achieve optimal esthetic outcomes. This approach did not hinder early detection of local recurrence or affect clinical outcomes in this study.

SCC was larger than BCC both clinically and pathologically, while BSC was larger than BCC only upon microscopic examination. This clinical underestimation of BSC size might be related to the authors’ awareness of misdiagnosis before referral and biopsy results obtained in the tertiary hospital setting.

Only three studies have presented the risk factors for aggressive behaviors in BSC patients. The risk factors are margin involvement, male gender, perineural invasion [36], tumor depth and size (T classification), incomplete resection, localization at the ear, deep maximal vertical infiltration, muscle and vessel invasion [28], a maximum lesion diameter  >3.0 cm, and perineural and lymphatic invasion [26].

In this study, only one distant metastasis occurred after WLE without any adjuvant radiation or chemotherapy during a median follow-up period of 96 months. To compare the outcomes of the three tumor groups, the authors used Kaplan–Meier survival analysis with the log-rank test to analyze the time–event relationship in the study. Significant differences were observed among the three groups for local recurrence (*p* = 0.019) and total adverse events (*p* = 0.046). However, statistical significance from the log-rank test was lost after multivariate adjustment on Cox regression analysis (BSC vs. BCC: *p* = 1.000 and *p* = 0.953 for local recurrence and total adverse events, respectively; BSC vs. SCC: *p* = 0.629 and *p* = 0.737, respectively). As a result, BCC without any adverse event showed the most promising disease-free outcome; BSC had a tendency to occur more frequently in total adverse events and SCC had a tendency to occur in local recurrence and total adverse events (Figure 2).

In recent studies applying the narrowest definition of BSC [18,24,29,31,33], less aggressive behavior was observed for BSC compared to SCC with low local relapse rate (0–4.1%) and no evidence of regional and/or distant metastasis (Table 1). Some older studies reported its aggressiveness to be the same as (local relapse rate 4.5–12.1%, lymph node metastasis rate 5.6–6.1%, distant metastasis rate 2.2%) [28,38] or even more aggressive than (local relapse rate 29–45.7%, lymph node metastasis rate 8.6–18%, distant metastasis rate 0–7.4%) [35,36,39] SCC, which typically has a 5-year recurrence rate of 8% for low-risk lesions treated by WLE and 3% for high-risk lesions treated by MMS and a metastatic rate of 0.5–6% [48]. In particular, studies featuring the broadest or modest definitions of BSC had the tendency to report a much higher local recurrence rate (8.9–31.4%), regional lymph node metastasis rate (0–14.3%), and distant metastasis rate (0–3%) [4,21,23].

In contrast to the forementioned studies, our analysis yielded mixed findings, with a clearly significant overall log-rank test but inconclusive pairwise Cox regression results between BSC and the other two tumors. In the authors’ opinion, such ambiguous findings may be attributable, in order of presumed importance, to the insufficient sample size (particularly only twenty BSC cases was included) and low statistical power; high censoring rates (BSC 30.0%, BCC 44.6%, and SCC 46.9%) with potential distortion of estimates; the use of newer statistical methods for BSC; ethnic, temporal, and community-level differences; and differences in stage distribution.

Among these five possibilities, the limited sample size reflected the real-world constraints, including the low incidence of BSC and even rarer occurrences of adverse events. High censoring rates observed across all three groups may be explained by multiple comorbidities in elderly patients (unrelated deaths: 15.0%, 9.2%, and 13.6% for BSC, BCC, and SCC, respectively), as well as the relatively favorable prognosis that has become increasingly recognized in recent years (follow-up loss: 15.0%, 35.4%, and 33.3% for BSC, BCC, and SCC, respectively).

To our knowledge, Kaplan–Meier survival analysis with the log-rank test and Cox regression analysis were applied to BSC for the first time in this study in order to mitigate the potential overestimation inherent in previous analyses based solely on Chi-square test or Fisher’s exact tests. Kaplan–Meier analysis is specifically designed for time-to-event data and is superior to methods using a contingency table, which consider only cumulative proportions at fixed time points. In fact, when we applied pairwise Fisher’s exact test to the outcome parameters in this study, we observed statistically significant differences between BCC and SCC in terms of local recurrence and total adverse events (both *p* = 0.017).

Contrary to studies in Caucasian populations, where significant prognostic differences between BCC and SCC have been consistently reported [49,50], recent large-scale prognostic studies in Korea have not demonstrated significant differences between BCC and SCC [51,52]. In a nationwide population-based study, the 5-year relative survival rate of SCC improved from 77.3% in 1996–2000 to 89.3% in 2015–2019, whereas that of BCC remained relatively stable, from 101.0% to 103.3% over the same period [52]. These findings suggest that ethnic (Asian), temporal, and community-level factors (e.g., early detection and advanced treatment under a national health insurance system) may contribute to the observed differences.

There were no significant differences in stage distribution according to AJCC-8 clinical T staging, BWT T staging, and AJCC-8 pathological T staging. BSC cases were classified into more advanced stages than BCC according to NCCN classification; however, this difference would be expected to reinforce the distinction between the two groups rather than contribute to the mixed results.

In this Asian cohort, BSC did not consistently show a clearly worse prognosis than BCC or SCC, which contrasts with Caucasian population reports. These findings suggest that the prognostic behavior of BSC may vary by population and clinical context, and that the traditionally held view of BSC as a uniformly high-risk disease may not be universally applicable. However, the small number of BSC cases, limited statistical power, and high censoring rates warrant cautious interpretation of these results and preclude definitive prognostic conclusions.

In this study, the authors performed conventional WLE as the sole surgical treatment without any adjuvant procedure; however, we could obtain satisfactory results. One pulmonary metastasis occurred in one patient with a size of 85 mm and a thickness of 7 mm pathologically (110 mm size clinically). A BCC of this size (over 5 cm) has a metastatic rate of at least 25% [53,54]. Extrapolating from the most recent NCCN guidelines for BCC and SCC, this very-high-risk patient may be an appropriate candidate for intraoperative SLNB, postoperative adjuvant radiation and chemotherapy [8,9].

Satisfactory outcomes were probably due to the application of a sufficient safety margin and double histopathologic check-up of IOFS and POPS. Although MMS is a standard surgical method for high-risk BCC and SCC [8,9,48], the authors believe that WLE could be a successful surgical option in BSC, as proved in some authors’ results [19,22,26]. Especially in elderly morbid patients with limited economic resources, this option might bring benefits of a relatively short operation time and lower cost. Although other studies claimed superior local control results of MMS compared to WLE in BSC [21,33], their research is based on an indirect narrative interstudy comparison with the abovementioned older literature as a form of expert opinion [35,36,39]. Only heterogeneous samples are provided in terms of primary or recurrent cases and risk factors. There is still no article in which direct prospective or retrospective intra-study comparisons have been made between the results of MMS and WLE for BSC. In fact, probably because of their rarity, inattention and underreporting, many studies take the form of case reports, and only two articles were found to be prospective in their design. Most retrospective intra-study comparisons were between BSC, BCC and SCC (Table 1).

This study has obvious limitations, including a small sample size, a high censoring rate and the retrospective study design. The true biological nature of BSC should be investigated with a large multi-center prospective or cohort study in the future.

## 5. Conclusions

In this Asian cohort, BSC has a trend toward higher rates of overall adverse outcomes compared to BCC, although this difference did not reach definitive statistical significance, unlike the findings reported in Caucasian populations. Early detection and appropriate treatment at the individual patient level are warranted to minimize rare but clinically relevant adverse events and reproduce favorable outcomes at the population level. Wide local excision followed by local flaps could be a successful surgical option with an adequate safety margin and double histopathologic intraoperative and postoperative check-up.

## Figures and Tables

**Table 2:** Demographic characteristics of BSC, BCC and SCC (in parentheses, the percentages are indicated, unless data are presented as ranges).

	BSC	BCC	SCC	Total †	Significance ‡
No. of patients	20 (9.8%)	120 (58.8%)	68 (33.3%)	204 (101.9%)	
No. of lesions	20 (8.7%)	130 (56.3%)	81 (35.0%)	231 (100%)	
Age, median (range)	72.5 (50–90)	72.5 (32–91)	78.5 (34–96)		BSC and BCC patients were younger than SCC patients
Gender					
male	8 (40.0%)	47 (39.2%)	23 (33.8%)		
female	12 (60.0%)	73 (60.8%)	45 (66.2%)		

† One BSC patient had a concomitant SCC. Three patients had both BCC and SCC simultaneously. ‡ *p* values < 0.05 were considered statistically significant for overall comparisons among the three groups. When a significant overall difference was detected, post hoc pairwise analyses were performed with Bonferroni correction, and statistical significance was set at *p* < 0.017 (0.05/3).

**Table 3:** Clinical characteristics of BSC, BCC and SCC (In parentheses, the percentages are indicated unless presented as ranges) ＊.

	BSC	BCC	SCC	Significance ‡
Location				
head and neck #	18 (90.0%)	119 (91.5%)	69 (85.2%)	
Trunk	1 (5.0%)	8 (6.2%)	6 (7.4%)	
Extremities	1 (5.0%)	3 (2.3%)	6 (7.4%)	
Duration, years, mean ± SD	4.7 ± 3.5	3.3 ± 4.9	2.0 ± 2.2	longer duration of BSC, BCC
Recurrent cases	2 (10.0%)	21 (16.2%)	12 (14.8%)	
Size (mm), median (range) $	12.5 (6–110)	10.0 (3–35)	18.0 (7–135)	SCC > BCC
Clinical findings				
unclear border	16 (80.0%)	19 (14.6%)	13 (16.0%)	more irregular borders in BSC
pearly appearance	1 (5.0%)	12 (9.2%)	0 (0%)	more glistening BCC than SCC
pigmentation	14 (70.0%)	90 (69.2%)	31 (38.3%)	more pigmented BCC than SCC
erythema	3 (15.0%)	16 (12.3%)	22 (27.2%)	more erythema in SCC than BCC
ulcer	5 (25.0%)	14 (10.8%)	18 (22.2%)	
cT staging on AJCC-8 #				higher cT staging of SCC than BCC
cT1	15 (83.3%)	111 (93.3%)	49 (71.0%)	
cT2	2 (11.1%)	8 (6.7%)	14 (20.3%)	
cT3	1 (5.6%)	0 (0%)	6 (8.7%)	
cT4	0	0	0	
head and neck total #	18 (100%)	119 (100%)	69 (100%)	
NCCN classification				higher NCCN of BSC, SCC than BCC
Low-risk lesions	0	5 (3.8%)	2 (2.5%)	
High-risk lesions	15 (75.0%)	125 (96.2%)	58 (71.6%)	
Advanced or				
very-high-risk lesions ∫	5 (25.0%)	0	21 (25.9%)	
BWH T staging ∬				
T1	14 (70.0%)		58 (74.3%)	
T2a	3 (15.0%)		13 (16.7%)	
T2b	3 (15.0%)		6 (7.7%)	
T3	0 (0%)		1 (1.3%)	
Immunosuppression	1 (5.0%)	4 (3.3%)	0 (0%)	

＊ AJCC-8, American Joint Committee on Cancer, 8th edition; NCCN, National Comprehensive Cancer Network; BWH T staging, Brigham and Women’s Hospital tumor staging. ‡ *p* values < 0.05 were considered statistically significant for overall comparisons among the three groups. When a significant overall difference was detected, post hoc pairwise analyses were performed with Bonferroni correction, and statistical significance was set at *p* < 0.017 (0.05/3). # In the AJCC-8 classification, NMSCs are staged for head and neck lesions only. $ Clinical size, not pathological size. ∫ Advanced lesions for BCC and very-high-risk lesions for SCC and BSC. Advanced BCC means locally advanced, nodal metastases, or distant metastases cases when surgery and/or radiation therapy are not curative according to NCCN. National clinical practice guidelines in oncology: Basal cell skin cancer (version 1.2026). ∬ BWH T staging was devised for cSCC only, not for BCC.

**Table 4:** Therapeutic characteristics of BSC, BCC and SCC (in parentheses, the percentages are indicated, unless data are presented as ranges).

	BSC	BCC	SCC	Significance ‡
Safety margin (mm), mean ± SD	6.6 ± 3.9	4.4 ± 1.3	8.5 ± 6.1	SCC > BSC > BCC
Initial resection attempts, mean ± SD ∬	1.3 ± 1.2	1.1 ± 0.3	1.1 ± 0.3	
Once	17 (89.5%)	114 (95.0%)	64 (90.1%)	
Twice	1 (5.3%)	3 (2.5%)	7 (9.9%)	
over three times	1 (5.3%)	3 (2.5%)	0 (0.0%)	
No. of operations, mean ± SD ∆	1.1 ± 0.2	1.0 ± 0.0	1.1 ± 0.4	
Once	19 (95.0%)	118 (100%)	63 (95.5%)	
Twice	1 (5.0%)	0 (0%)	2 (3.0%)	
over three times	0 (0.0%)	0 (0%)	1 (1.5%)	
Operation time (minutes), mean ± SD	77.5 ± 34.9	68.5 ± 34.3	96.8 ± 65.6	BSC, SCC > BCC
Anesthesia				
General	2 (10.0%)	3 (2.5%)	14 (20.3%)	SCC > BCC
Local	18 (90.0%)	117 (97.5%)	55 (79.7%)	BCC > SCC
Reconstruction				less PC in BSC than BCC
local flap	10 (50.0%)	40 (31.5%)	23 (28.4%)	
regional flap	1 (5.0%)	1 (0.8%)	0	
distant flap, forehead	0	2 (1.6%)	0	
distant flap, free flap	0	0	1 (1.2%)	
myocutaneous/fasciocutaneous	0	0	4 (4.9%)	
FTSG	6 (30.0%)	48 (37.8%)	29 (35.8%)	
STSG	2 (10.0%)	5 (3.9%)	8 (9.9%)	
local flap + FTSG #	1 (5.0%)	1 (0.8%)	1 (1.2%)	
local flap + STSG $	0	0	1 (1.2%)	
primary closure	0	30 (23.6%)	14 (17.3%)	
Adjuvant therapies	0 (0%)	0 (0%)	RT 2 (2.5%)	

# FTSG, full-thickness skin graft. $ STSG, split-thickness skin graft. ‡ *p* values < 0.05 were considered statistically significant for overall comparisons among the three groups. When a significant overall difference was detected, post hoc pairwise analyses were performed with Bonferroni correction, and statistical significance was set at *p* < 0.017 (0.05/3). ∬ Initial resection attempts refer to the number of resection attempts needed for negative frozen results at the first operation. ∆ No. of operations refers to the total number of operations needed for tumor eradication.

**Table 5:** Histological characteristics of BSC, BCC and SCC (in parentheses, the percentages are indicated, unless data are presented as ranges) ＊.

	BSC	BCC	SCC	Significance ‡
Correct initial diagnosis ※	5 (25.0%)			
Size (mm), median (range) $	10.0 (2–85)	6.0 (0.3–32)	10.8 (1–130)	BSC, SCC > BCC
Thickness (mm), mean ± SD	2.7 ± 2.0	2.5 ± 1.9	3.7 ± 3.7	
MVI				
S (superficial: dermis)	15 (75.0%)	93 (86.1%)	57 (80.3%)	
M (medium: SQ)	4 (20.0%)	13 (12.0%)	11 (15.5%)	
D (deep)	1 (5.0%)	2 (1.9%)	3 (4.2%)	
Clark level				
II	0	3 (2.8%)	8 (11.9%)	
III	3 (15.0%)	40 (37.4%)	24 (35.8%)	
IV	12 (60.0%)	50 (46.7%)	20 (29.9%)	
V	5 (25.0%)	14 (13.1%)	15 (22.4%)	
PNI	2/20 (10.0%)	1/116 (0.86%)	2/74 (2.7%)	BSC > BCC
LVI	0	0	1/75 (1.3%)	
R classification				
R0	19 (95.0%)	114/122 (93.4%)	66/66 (100%)	
R1/R2	1 (5.0%)	8/122 (6.6%)	0/66 (0%)	
Free margin				
lateral (mm), mean ± SD	4.3 ± 2.9	3.0 ± 1.3	5.4 ± 3.8	BSC, SCC > BCC
deep (mm), mean ± SD	2.3 ± 1.5	2.2 ± 2.0	2.7 ± 2.1	
pT staging on AJCC-8 #				
pT1	15 (83.3%)	110 (92.4%)	56 (81.1%)	
pT2	0 (0%)	1 (0.9%)	4 (5.8%)	
pT3	3 (16.7%)	8 (6.7%)	9 (13.1%)	
pT4	0	0	0	
head and neck total #	18 (100%)	119 (100%)	69 (100%)	

＊ MVI, maximal vertical infiltration; PNI, perineural invasion; LVI, lymphovascular invasion. $ Pathological size, not clinical size. ‡ *p* values < 0.05 were considered statistically significant for overall comparisons among the three groups. When a significant overall difference was detected, post hoc pairwise analyses were performed with Bonferroni correction, and statistical significance was set at *p* < 0.017 (0.05/3). ※ Preoperative one-sample punch biopsy at the office revealed twelve BCCs, one actinic keratosis, two SCCs, and five BSCs as the initial diagnoses. # In the AJCC-8 classification, NMSCs are staged for head and neck lesions only.

**Table 6:** Outcome characteristics of BSC, BCC and SCC (in parentheses, the percentages are indicated, unless data are presented as ranges) ＊.

	BSC	BCC	SCC	Significance ‡
Follow-up (months), median (range)	96.0 (1–182)	54.0 (1–190)	40.0 (1–175)	BSC > BCC, SCC
Follow-up more than 60 months #	15/20 (75.0%)	61/120 (50.8%)	33/68 (48.5%)	
Local recurrence	0/16 (0%)	0/61 (0%)	4/36 (11.1%)	loss of significance after Cox ∆
Lymph node metastasis	0/16 (0%)	0/61 (0%)	2/36 (5.6%)	
Distant metastasis	1/16 (6.3%)	0/61 (0%)	1/36 (2.7%)	
Total no. of adverse outcomes	1/16 (6.3%)	0/61 (0%)	4/36 (11.1%)	loss of significance after Cox ∆

＊ Including three forms of aggressive behavior cases, the patients in the denominator totaled 16 (16/20 = 80.0%), 61 (61/120 = 50.8%), and 36 (36/68 = 52.2%). ‡ For the follow-up period, *p* values < 0.05 were considered statistically significant for overall comparisons among the three groups. When a significant overall difference was detected, post hoc pairwise analyses were performed with Bonferroni correction, and statistical significance was set at *p* < 0.017 (0.05/3). Kaplan–Meier analysis was used alongside the log-rank test to estimate disease-free survival outcomes among the three groups. When an overall significant difference was detected (*p* < 0.05), pairwise comparisons between the two groups were performed using Cox proportional hazards regression, and statistical significance for these pairwise tests was set at *p* < 0.017 (0.05/3). # Patients followed up for at least 60 months or who presented with local relapse, lymph node metastasis or distant metastasis were counted; Among one BSC and four SCC patients with adverse outcomes, one SCC patient with local relapse only without lymph node or distant metastasis were successfully treated with a second wide local excision and remained disease-free for 102 months. Another single BSC and three SCC patients were lost for follow-up. ∆ Statistical significance from the log-rank test (*p* = 0.019 for local recurrence; *p* = 0.046 for total adverse events) was lost after multivariate Cox proportional hazards regression adjustment (BSC vs. BCC: *p* = 1.000 and *p* = 0.953 for local recurrence and total adverse events, respectively; BSC vs. SCC: *p* = 0.629 and *p* = 0.737, respectively).

## Data Availability

Data are available on request due to ethical reasons. Patient’s records are kept in the archives of the Department of Plastic and Reconstructive Surgery, Chungbuk National University Hospital, Cheongju, Chungbuk, Republic of Korea.

## Supplementary Materials

The following supporting information can be downloaded at https://www.mdpi.com/article/10.3390/jcm15062449/s1, Table S1: Incidences of BSC, MBCC, BCC and SCC in six original articles reporting five BSC cases in the non-Caucasian population.
