# Supplementary material for: Basosquamous Cell Carcinoma: A Summary of the Definitions and Demographic, Clinical, Therapeutic, Histological, and Outcome Analysis of 20 Consecutive Basosquamous Cell Carcinomas in Comparison with 130 Basal Cell and 81 Squamous Cell Carcinomas in a Single Institution
Source: J Clin Med. 2026 Mar 23;15(6):2449. doi: 10.3390/jcm15062449 (PMC13027180; doi:10.3390/jcm15062449)
Supplement: Supplementary file 1 [file jcm-15-02449-s001.zip › jcm-4158012-supplementary.pdf]

Table S1. Incidences of BSC, MBCC, BCC and SCC in six original articles reporting five BSC cases in non-Caucasian population.

Reference/Authors, Year	Patients	BSC (%)	MBCC (%)	NMSC (as control) (%)
Song KY et al., 1994 [17]	213 BCCs	1 (0.5%)	9 (4.2%)	
Jeon YM et al., 1999 [16]	62 BCCs	1 (1.6%)		
Oh ST et al., 2005 [15]	87 BCCs	1 (1.1%)	11 (13.8%)	
Oh ST et al., 2006	same case as Oh ST et al., 2005 [15]			
Park CH et al., 2008 [13]	19 facial skin cancers	1 (5.3%)		11 BCCs (57.9%), 5 SCCs (26.3%)
Park HN et al., 2014 [12]	73 eyelid skin cancers	1 (1.4%)		41 BCCs (56.2%), 17 SCCs (23.3%)
Total BCCs [15-17]	362 BCCs	3/362 (0.8%)	20/362 (5.5%)	
Total facial skin cancers [12, 13]	92 facial skin cancers	2/92 (2.2%)		

The cumulative incidence rates of BSC in those six original articles were 0.8% (3/362 BCCs) to 2.2% (2/92 facial skin cancers) excluding one duplicate case in references 15 [12, 13, 15-17]. The incidence rates of MBCC at those articles were 4.2% (9/213 BCCs) [17] to 13.8% (11/87 BCCs) [15]. The incidence rates of BCC versus SCC at the same articles ranges from 2.2 (11/5) to 2.4 (41/17).
